# Computational Strategies for Broad Spectrum Venom Phospholipase A_2_ Inhibitors

**DOI:** 10.1021/acs.jcim.5c00045

**Published:** 2025-04-22

**Authors:** David A. Poole, Laura-Oana Albulescu, Jeroen Kool, Nicholas R. Casewell, Daan P. Geerke

**Affiliations:** † Department of Chemistry and Pharmaceutical Sciences, Amsterdam Institute for Molecular and Life Sciences, Vrije Universiteit Amsterdam, De Boelelaan 1105, Amsterdam 1081 HV, the Netherlands; ‡ Centre for Snakebite Research & Interventions, Liverpool School of Tropical Medicine, Pembroke Place, Liverpool L3 5QA, U.K.

## Abstract

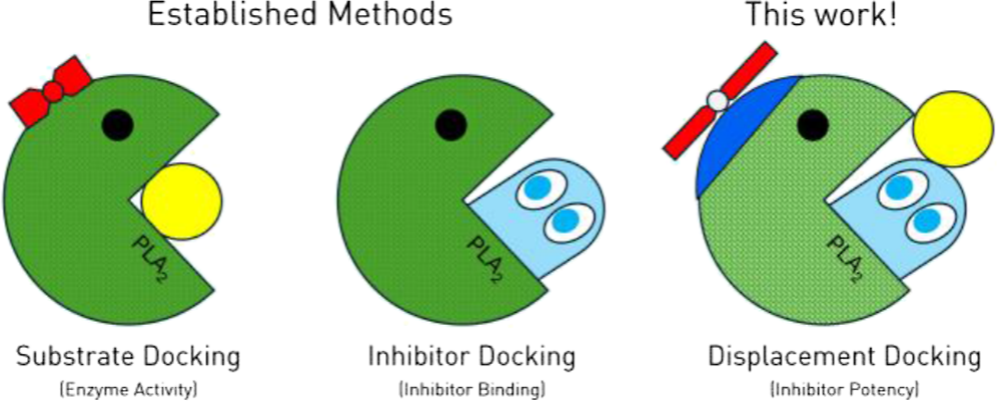

Snakebite envenoming is a persistent cause of mortality and morbidity worldwide due to the logistical challenges and costs of current antibody-based treatments. Their persistence motivates a broad interest in the discovery of inhibitors against multispecies venom phospholipase A_2_ (PLA_2_), which are underway as an alternative or supplemental treatment to improve health outcomes. Here, we present new computational strategies for improved inhibitor classification for challenging metalloenzyme targets across many species, including both a new method to utilize existing molecular docking, and subsequent data normalization. These methods were improved to support experimental screening efforts estimating the broader efficacy of candidate PLA_2_ inhibitors against diverse viper and elapid venoms.

## Introduction

**Figure 1:**
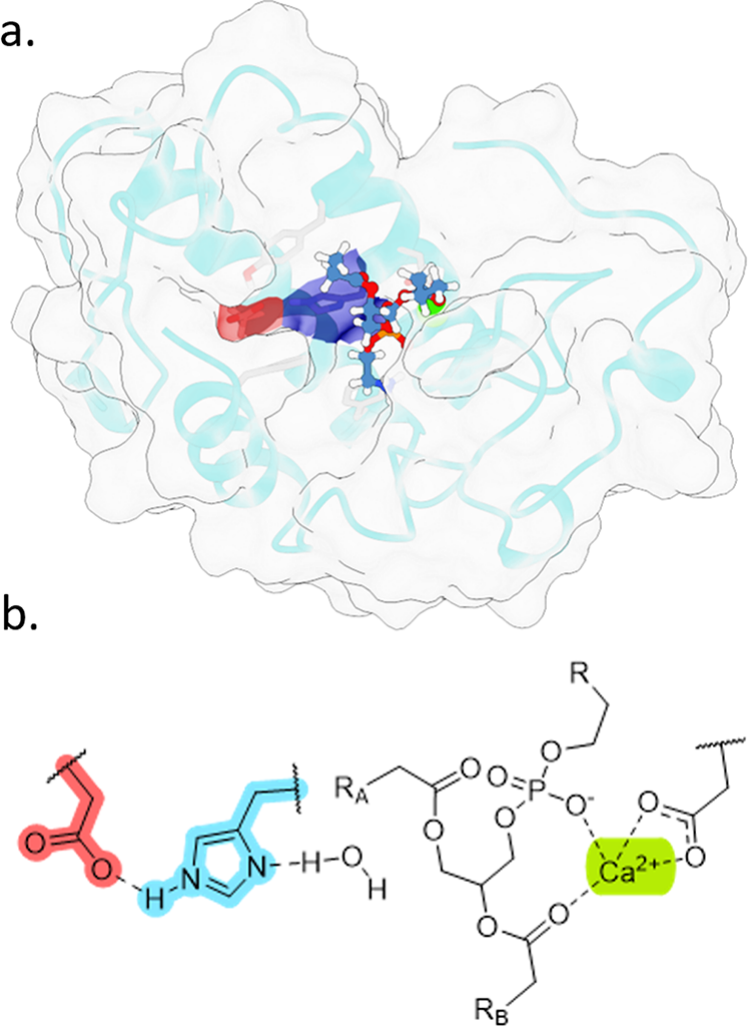
Rendering of a PLA_2_ (RCSB: 1TGM)^20^ featuring a phospholipid substrate docked at the active site (a), and depiction of the enzyme active state for phospholipid hydrolysis (b) with aspartate (red), histidine (blue), and calcium ion (green) highlighted.

Snakebite envenoming remains a critical health concern, particularly in developing regions with limited access to life saving care arising from unsolved logistical challenges.^1^ Current treatment relies on antivenom, a costly polyclonal antibody-based medicine derived from hyper-immunized animals that neutralizes specific venom toxins by binding to their unique surface epitopes.^2^ However, the variability of these epitopes means that antivenoms are often specific to only those venoms used in their manufacture.^3^ In addition, stocks of antivenom must be stored at low temperatures and regularly replaced due to their short shelf life, while the need for intravenous delivery and management of adverse reactions dictate that antivenoms must be given in a clinical environment, which delays treatment.^1,2^ In response, there is a growing interest among medicinal chemists in developing shelf-stable small-molecule inhibitors capable of neutralizing a broad spectrum of venom toxins.^4^ Such medicines could reduce the loss of life and limb by providing early community-level interventions soon after a bite. Among the most promising targets for these inhibitors are members of the enzymatic venom phospholipase A_2_ (PLA_2_) toxin family, a key mediator of venom toxicity.^4−6^ These functionally diverse enzymes facilitate the hydrolysis of phospholipid membranes (Figure 1) and some can lead to immediate tissue damage and necrosis at the wound site. This catalytic activity also releases arachidonic acid as a product, which leads to inflammation and systemic effects.^5^

Experimental approaches to inhibitor discovery also face logistical challenges. For example, limited availability of authentic venom samples from diverse species hinders both the identification and validation of candidate compounds.^6^ While efforts are widely made to overcome this through recombinant enzyme proteins, these logistical difficulties emphasize a need for efficient strategies to expedite the discovery of broad spectrum PLA_2_ inhibitors.

In this context, computational approaches such as molecular-docking based virtual screening offer a promising avenue for discovering new PLA_2_ inhibitors.^7^ Molecular docking is a widely used technique for screening compound libraries to identify potential ligands that bind to the enzyme active site.^8^ However, many widely available docking methods rely on affinity scores that do not accurately represent the coordination of inhibitors to metal centers such as those found in PLA_2_s.^9^ This challenge is further pronounced in the discovery of broad-spectrum inhibitors for PLA_2_ isoforms where subtle interspecies structural variances may limit inhibitor binding.^10^ These structural variances affect the affinity estimates from molecular docking, thus posing additional challenges in the classification of broadly acting inhibitors.

Here we report methodological advancements in the application of molecular docking to discover broad-spectrum inhibitors against snake venom PLA_2_s. We make use of experimental data for *Daboia russelii* PLA_2_ activity inhibition, from a drug-repurposing program for the treatment of snakebite envenoming.^11^ In the current work we present a complete workflow to identify PLA_2_ inhibitors, starting from structural studies of the enzymes found in viper and elapid snake families to an eventual assessment and comparison of inhibitor binding efficacy. Central to our approach is a novel technique called “displacement docking”, which evaluates the ability of a substrate to bind to the active site of an enzyme–inhibitor complex. This technique uses traditional docking methods as a means to assess how well a ligand obstructs the active site to hinder enzyme reactivity.^12^ We assessed the performance of traditional affinity-based scoring (traditional direct docking) and our displacement docking with a pool of 192 ligands with experimentally measured inhibition of *D. russelii* PLA_2_ activity (see Table S3 of the Supporting Information). Our workflow was expanded by a standardization scheme based on substrate affinity to allow for cross-species comparisons of inhibitor effectiveness against PLA_2_s from viper and elapid snake species with reported crystallographic structures. Using the scores obtained, we assessed the similarity of the enzymes based on their inhibitor binding, providing new avenues for the discovery of broad-spectrum inhibitors.

## Results and Discussion

### Variation in Sequence and Structure of Viper and Elapid PLA_2_s

**Figure 2:**
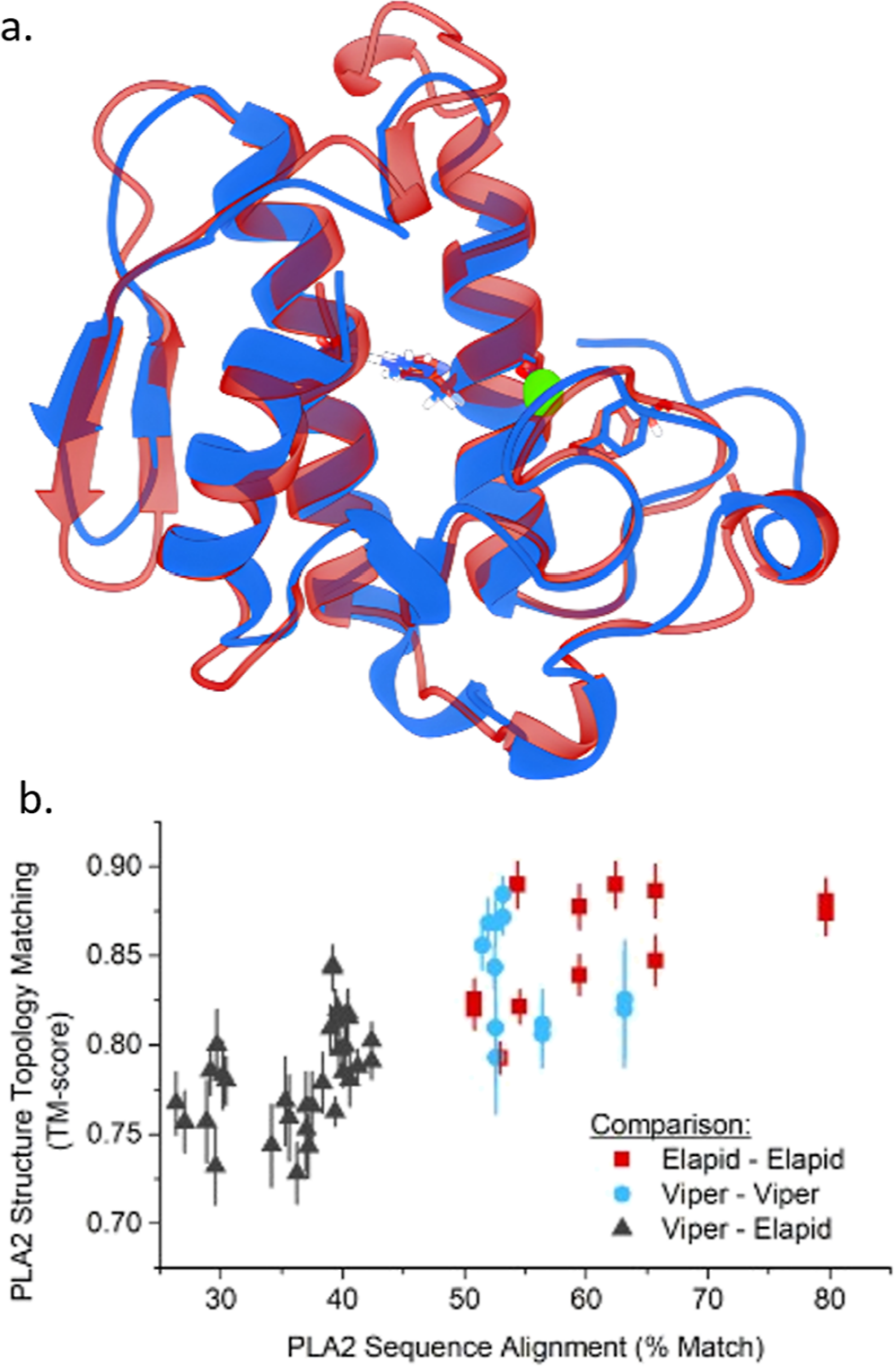
Structural comparison of common PLA_2_ isoforms: (a) renderings of aligned PLA_2_ structures from viper (blue) and elapid (red) snake families, and (b) correlation plot of sequence- and structure-based comparison metrics within (red and blue) or between (black) family groups (see Supporting Information Section S3 for methodological details). Error bars represent the interquartile range within the PLA_2_ structures.

Understanding the sequence- and structural-variability of PLA_2_s provides a basis for the structure-based discovery of broadly acting inhibitors. To quantify these variations, we first extracted crystallographic structures of eight venom PLA_2_ isoforms from the protein databank. These isoforms were derived from viper and elapid snake families and featured well-resolved Ca^2+^ active sites (Table 1).^13^ To better represent the solution state, the initial crystallographic structures were submitted to a standard molecular dynamics (MD) protocol (see Supporting Information Section S1 for methodological details), followed by hierarchical clustering to generate five representative structures for each PLA_2_.^14^ Sequence comparison quantifies the influence of evolutionary pressure and genetic drift on the primary amino-acid structure of an enzyme. In contrast, topological structure comparison with a template modeling score (TM-score) includes the structural changes in the protein backbone.^15^ Compared to primary sequence differences, this metric may be more sensitive to similar or conserved regions between proteins, such as enzyme active sites. To assess the evolutionary variation and structural similarity within and between viper and elapid PLA_2_s, we performed here pairwise sequence and structural comparisons using MaxCluster^16^ among our MD-derived structures (Figure 2).

**Table 1:** Crystal Structure Models Used in This Study

PDB ID	species	family	res. (Å)	ref
1OZ6	Echis carinatus	viper	2.60	(20)
1TGM	Daboia russelii	viper	1.86	(21)
1BJJ	Gloydius halys *A*^a^	viper	2.80	(22)
1PSJ	Gloydius halys *B*^a^	viper	2.00	(23)
1POA	Naja atra	elapid	1.50	(24)
1TD7	Naja sagittifera	elapid	2.50	(25)
1GP7	Ophiophagus hannah	elapid	2.60	(26)
1FE5	Bungarus caeruleus	elapid	2.45	(27)

a Divergent reported PLA_2_ variants.^21^

Despite a common functional nature of PLA_2_s, we found that the amino-acid sequences between viper and elapid isoforms were widely dissimilar (Match = 20–45%; Figure 2b, black). In contrast, the topological structures of these enzymes were broadly similar between the two snake families (TM-score = 0.70–0.85). We surmise this similarity reflects the consistent spatial arrangement of amino acids needed for substrate binding and catalytic activity, essential for the toxic biological role of many of these enzymes.^17,18^ The difference between these comparison metrics may reflect how the distant evolution of these enzymes permits significant sequential variability that undermines broad cross-species antivenom development, while retaining structural similarities that may be leveraged in the discovery of broad-spectrum inhibitors.

Comparisons of structures within the individual viper and elapid PLA_2_ families show stronger conservation of both the primary amino-acid sequence and enzyme topology (Figure 2b, red and blue), indicative of their independent evolutionary origins for roles in venom. However, from the inclusion of two PLA_2_s derived from *Gloydius halys* (1BJJ and 1PSJ)^22,23^ we found surprising insight into the sequence and structure differences between enzymes derived from a single venom mixture. Notably, these isoforms were widely dissimilar in amino-acid sequence (52%) compared to other isoform pairs while retaining consistent topological similarity (0.85). Here, the observed sequence variability is in line with reports of venom enzyme evolution^19^ and underscores a trend in PLA_2_s where variation exists amid a strong conservation of an essential enzyme topology, within even a single species. This additionally highlights the need to discover inhibitors against PLA_2_s that leverage common structural interactions.

### Development of a Candidate Inhibitor Protomer Library

**Figure 3:**
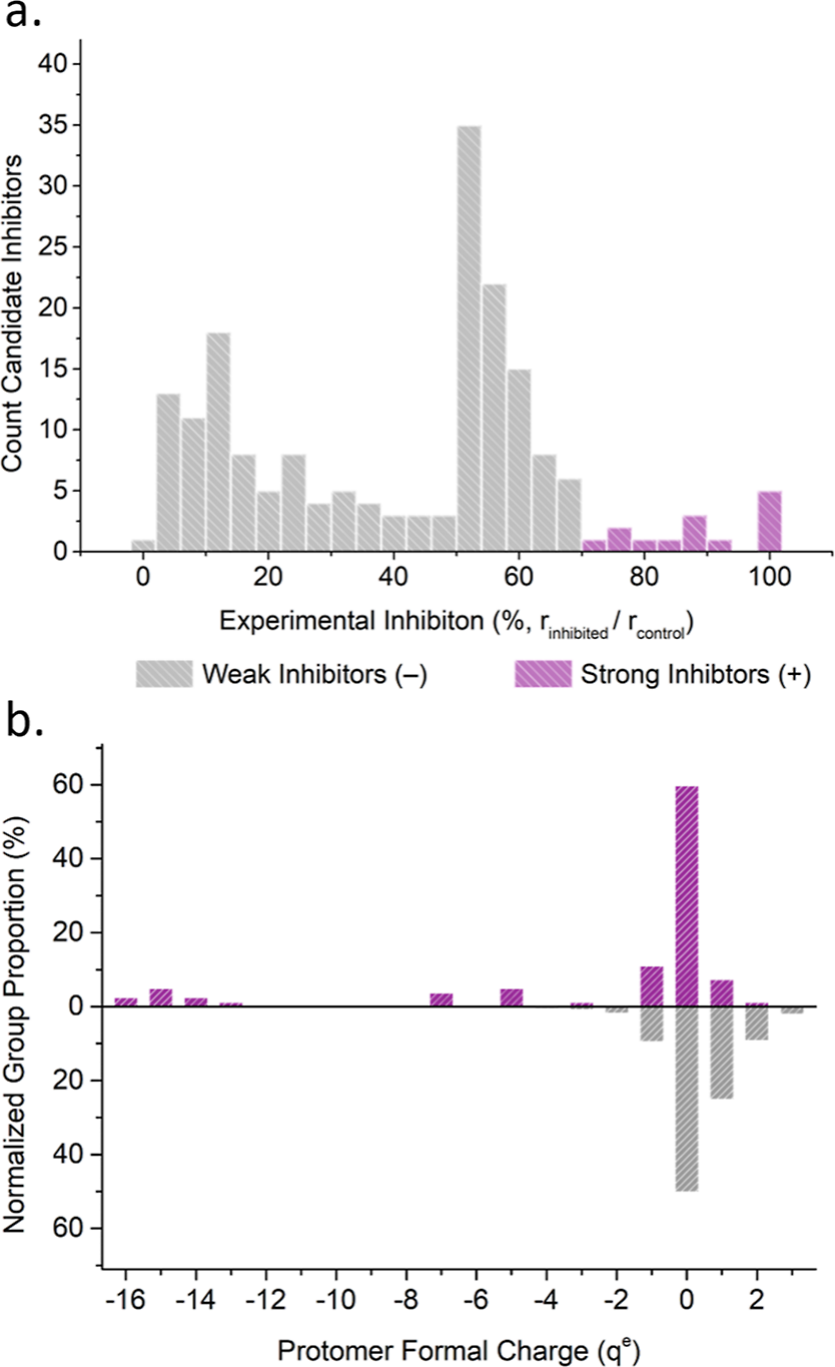
Distributions of candidate inhibitors by experimental inhibition (a, *n*_candidates_ = 192), and protomer charge (b, *n*_protomers_ = 961). We note that protomers from acidic polyphenols (e.g., tannic acid), were enumerated as highly charged species from Dimorphite-DL. Further details the prevalence of common drug-likeness parameters in candidate inhibitors are shown in Figure S2.

As a next step in our workflow development for structure-based identification of broad-spectrum PLA_2_ inhibitors, we prepared a library of 192 candidate molecules ranging from strong inhibitors of *D. russelii* PLA_2_ activity to noninhibitors assessed via high-throughput screening experiments (see Supporting Information Section S4 and Table S3).^11^ This initial screening focused on inhibition of PLA_2_ activity from *D. russelii* venom (1TGM) using a fixed inhibitor concentration (10 μM) for therapeutic relevance. By comparison to untreated control samples, inhibition was quantified as a percentage reduction in enzyme activity upon ligand addition and 70% inhibition was introduced as a cutoff to distinguish strong inhibitors from weak ones (Figure 3a). Since many candidates contain one or more acidic and/or basic moieties, their protonation state is expected to play a significant role in binding interactions with the PLA_2_ active site. Because binding activity may alter the protonation states, the protomers of each candidate inhibitor was enumerated from its SMILES representation using Dimorphite-DL within a wide pH range (5.0–8.0).^28^ This expanded library contained 961 protomers for the 192 candidates, with each protomer representing a protonation state attainable under experimental conditions (Table S4). Initial analysis of these protomers revealed a clear relationship between inhibition and charge, with a significant occurrence of highly charged compounds among the strong inhibitors (Figure 3b).

From the experimental high-throughput screening, the majority of candidates failed to fully inhibit *D. russelii* PLA_2_ activity (i.e., inhibition <100%, Figure 3a). However, 15 candidates surpassed the cutoff value of 70% inhibition as a threshold for therapeutically relevant hits. These 15 compounds were classified in this work as “true positives” for docking performance evaluation, while the remaining 178 were categorized as “true negatives”. Notably, most strong inhibitors contained one or more anionic carboxylic and/or phenolic acid moieties (Figure 3b). This trend likely reflects a strong association of these moieties to the cationic PLA_2_ active site. The clear bias toward negative charges in successful inhibitors under physiological pH values emphasizes the importance of accounting for deprotonation in our discovery approach. Additionally, these findings align with the broader trends in metalloenzyme inhibitor design where oxyacid moieties often demonstrate strong inhibition by metal-ion coordination.^29^

### Scoring Function Selection Based on Traditional Ligand Docking

**Figure 4:**
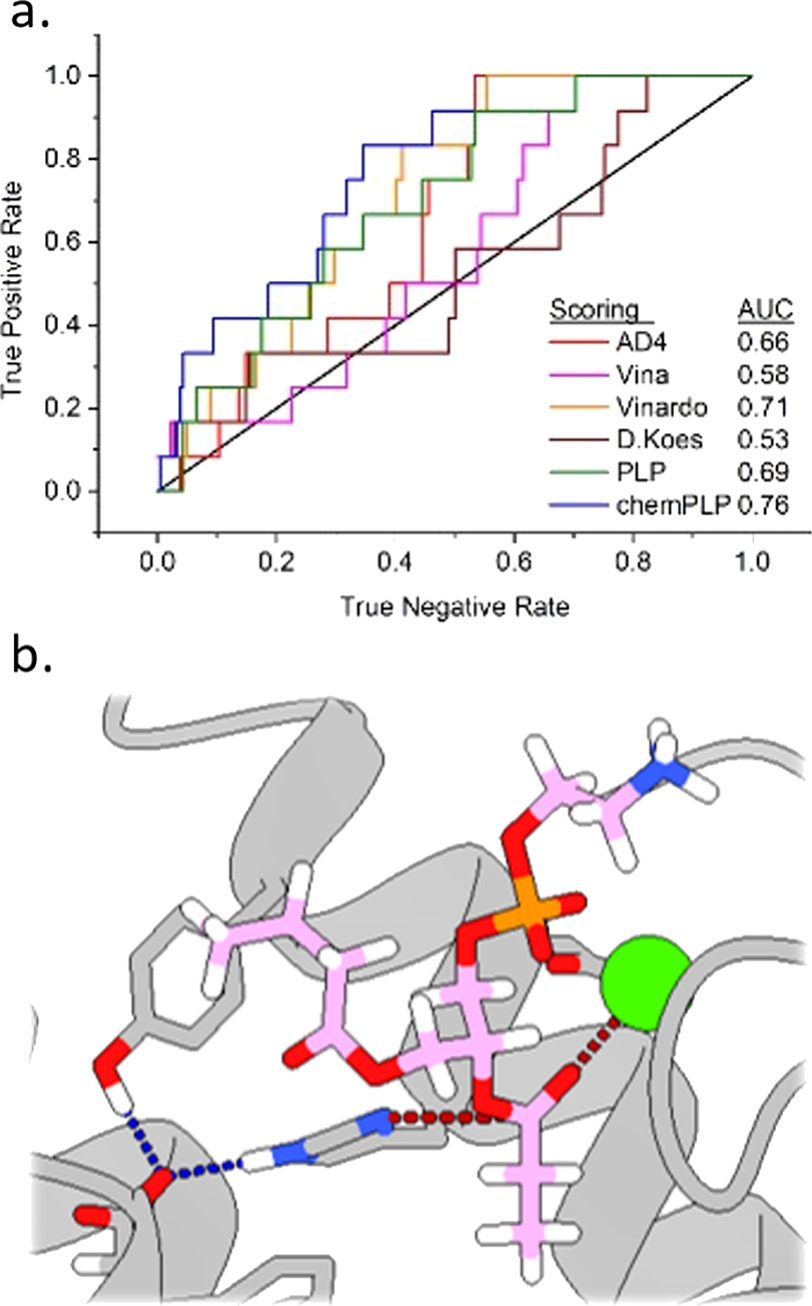
(a) Receiver operating characteristic (ROC) plot ordering of strong PLA_2_ inhibitors with different scoring functions^29,30^ (Table S4) and associated area-under-the-curve values (AUC); (b) the **S**^**M**^–1TGM complex from PLANTS1.2/*chemPLP*, showing the proton transfer hydrogen bonds (blue dashed lines) and relevant distances between the substrate and active site (red dashed lines).

To optimize molecular docking, we evaluated the performance of different scoring functions available in two docking programs: PLANTS1.2 (*PLP*, *chemPLP*)^30^ and smina (*AD4*, *vina*, *vinardo*, *dkoes*).^31^ Each scoring function was used with standard parameters (Table S4) to assess the affinity of protomers against the ensemble of *D. russelii* PLA_2_ structures (1TGM). Recognizing that PLA_2_ binding may influence the protonation state of the inhibitors,^32^ candidates were assigned the best score achieved from any protomer docked against any of the enzyme structures obtained from MD and clustering. The effectiveness and accuracy of the scoring functions were then assessed quantitatively with a receiver operating characteristic (ROC) plot, and qualitatively from the best pose found for 2-aminoethyl (2,3-bis(butyryloxy)propyl) phosphate (**S**^**M**^) used here as a model phosphatidylethanolamine substrate, Figure 4.

The performance of a classification metric, such as a scoring function, is readily evaluated by its ability to differentiate true positives (strong inhibitors) and true negatives (i.e., weak inhibitors) when ranked by the metric. In a ROC plot (Figure 4a), the area under the curve (AUC) provides a measure of the scoring function performance, where an AUC of 1.0 represents a perfect classification, and 0.5 corresponds to a random selection. The considered scoring functions that incorporate an electrostatic term, AD4 (AUC = 0.66) and DKoes (AUC = 0.53), were expected to perform well given the strong interaction of anionic inhibitors to the cationic active site. However, their lower AUC values suggest that their electrostatic or charge-dependent desolvation terms may lead to weak prediction of the binding strength of (poly)anionic species.^33^

Consistent with previous reports,^9^ both *PLP* (AUC = 0.69) and *chemPLP* (AUC = 0.76) outperform *vina* (AUC = 0.58) for metal–ligand interactions. While *vinardo* (AUC = 0.71) makes some improvement compared to *vina* (probably due to atom-type revisions^30,33,34^), qualitative assessment of a docked **S**^**M**^ suggests that phosphate parameters are deficient to describe correct coordination geometry (Figure S1). In contrast, docked poses selected with the *chemPLP* scoring function exhibit the expected activation of **S**^**M**^ through coordination of the phosphate and carbonyl oxygens and calcium (Figure 4b). From these assessments, *chemPLP* is here preferred as the most effective for PLA_2_ inhibitor discovery.

### Displacement Docking Using Ligand Masks to Assess Active-Site Obstruction

**Figure 5:**
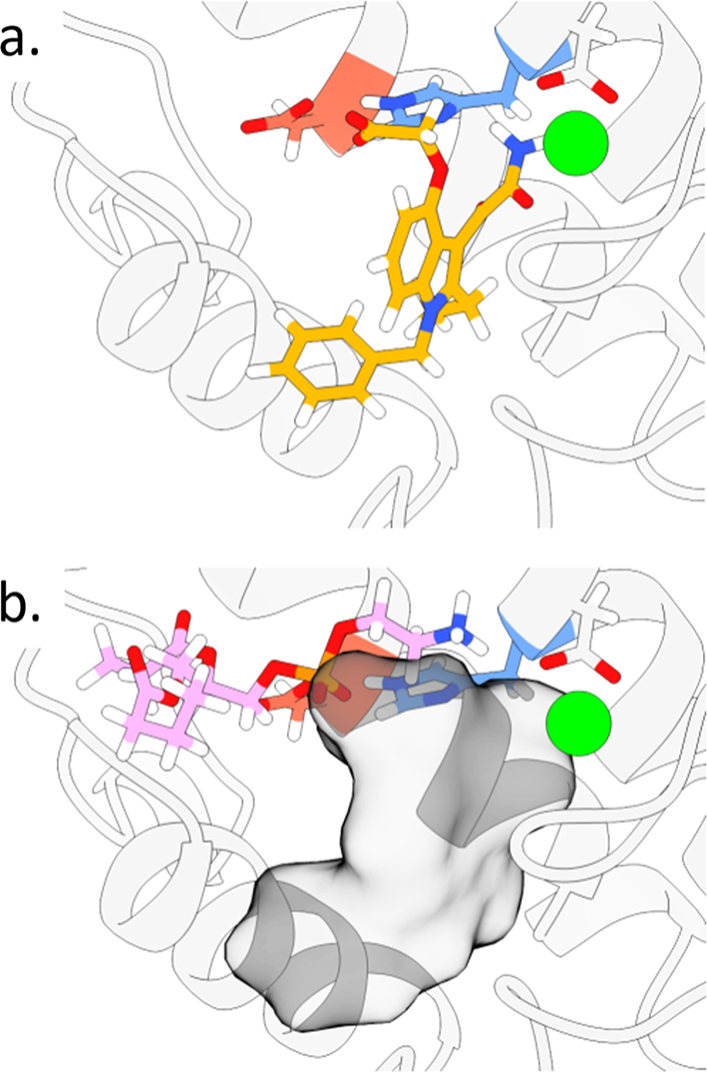
Visualization of (a) traditional and (b) displacement docking, with the ligand shown in orange (a), the penalty mask as a gray volume (b), and the substrate **S**^**M**^ (docked in the displacement docking step) in pink.

Displacement docking evaluates the ability of a substrate to bind to the active site of an enzyme–inhibitor complex by using traditional direct docking methods (Figure 5). In this process, the (predocked) inhibitor is treated as a mask with a strong penalty (Supporting Information Table S6) in order to exclude substrate-inhibitor interactions. The resulting displacement-docking score reflects the catalytically relevant active-site obstruction by the inhibitor.

**Figure 6:**
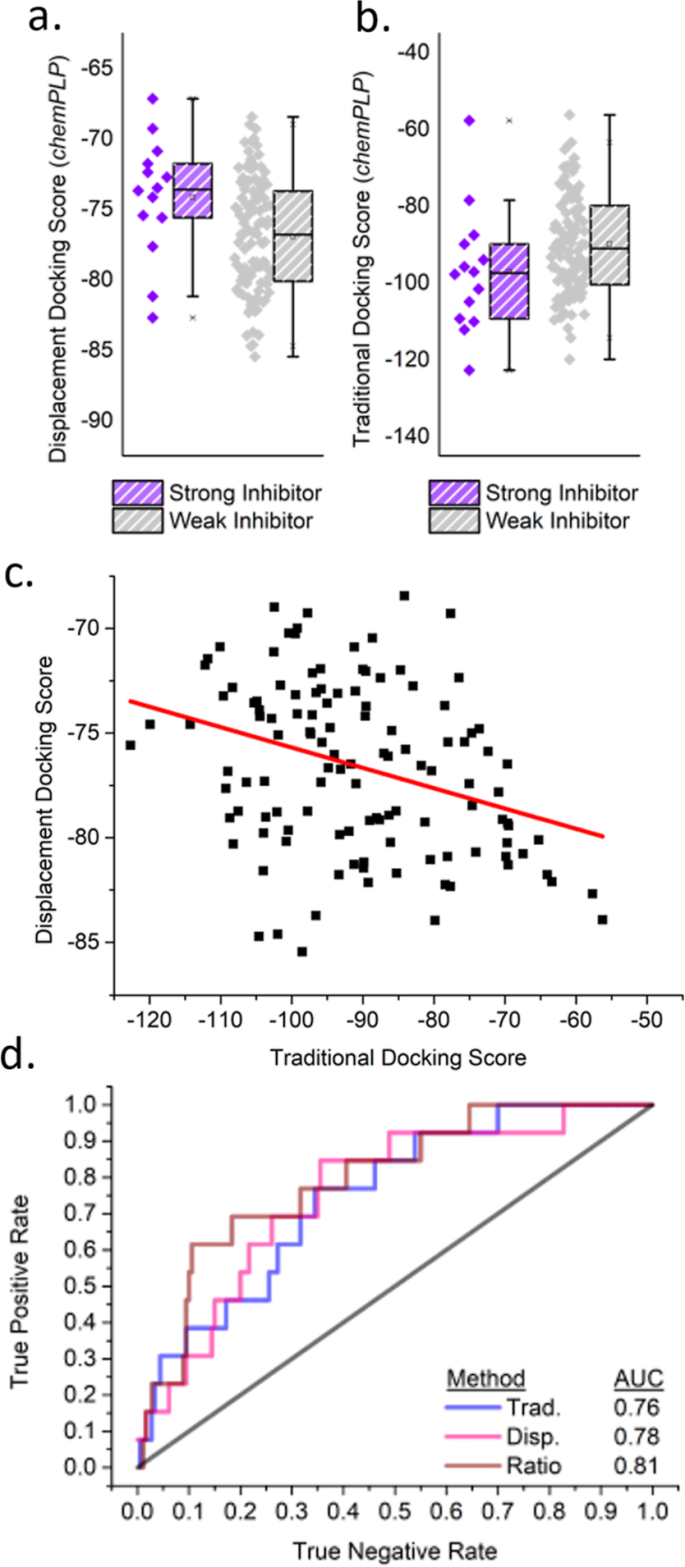
Distribution of scores (with boxes indicating the interquartile range of the data) for strong (purple) and weak (gray) inhibitors from either (a) displacement or (b) traditional direct docking approaches, as listed in Supporting Information Table S6. Their correlation is visualized by (c) a scatterplot with a linear fit (*R*^2^ = 0.09). Lastly, the performance of the two docking approaches alone or in combination as a ratio (eq 1) is assessed by (d) a ROC plot for the discovery of strong inhibitors against 1TGM, with AUC values indicated.

Intuitively, obstruction of the active site by an inhibitor reflects its potency. To evaluate the performance of displacement docking, we used substrate **S**^**M**^ with the enzyme–inhibitor complexes as templates that were previously obtained from traditional inhibitor docking into the *D. russelii* PLA_2_ structures (Figure 4). As with traditional direct docking, we selected after displacement docking the best score found across any of the obtained complexes as the score of the inhibitor. We then assessed the efficacy of our displacement-docking scoring approach to identify strong inhibitors from our candidate library, and the extent these scores are independent of traditional docking results (Figure 6).

Since the displacement-docking score reflects substrate binding, a more positive value indicates weaker substrate binding and hence stronger inhibition by the considered inhibitor, due to greater active-site obstruction. Although the differences between strong and weak inhibitors are found to be statistically significant for both docking methods, following a *t*-test displacement docking (*p* < 0.01, Figure 6a) gives a more distinct separation than traditional direct docking (*p* < 0.03, Figure 6b). Despite the individual performance of each method, we found minimal correlation (*R*^2^ < 0.1) between their results, indicating that molecular features promoting inhibitor binding may not necessarily obstruct substrate access (Figure 6c). The lack of correlation suggests that traditional and displacement docking scores could offer complementary insights, while potentially improving inhibitor classification. This was explored using a ratio of the traditional and displacement scores (eq 1), which as a metric would increase with both stronger binding interactions and more pronounced active site obstruction.
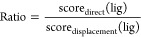
1

We compared the overall performance of displacement docking and the ratio metric using the ROC (Figure 6d) and found modest improvements (AUC = 0.78) over the traditional method (AUC = 0.76). Introducing the ratio metric resulted in further improved classification (AUC = 0.81). Taken together these findings validate displacement docking as an independent tool that enhances inhibitor classification over traditional direct docking approaches.

### Point-Based Identification of Broad Spectrum PLA_2_ Inhibitors

The structural diversity among PLA_2_s complicates the direct comparison of docking scores across different isoforms, hindering the discovery of broadly acting inhibitors. Based on the premise that effective inhibitors must outcompete the substrate for active site binding, we developed a binary classification system using substrate **S**^**M**^ to set a performance cutoff value per target. In addition to the scoring metrics discussed above (i.e., scores obtained in traditional direct docking, in displacement docking, or from the ratio in eq 1), we analyzed the metal and steric interaction components of the *chemPLP* docking scores due to their essential roles in mediating substrate activation and substrate binding (Figure 1b). The performance of our substrate-based cutoff is calculated by the proportional enrichment of strong inhibitors (eq 2).
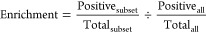
2

**Figure 7:**
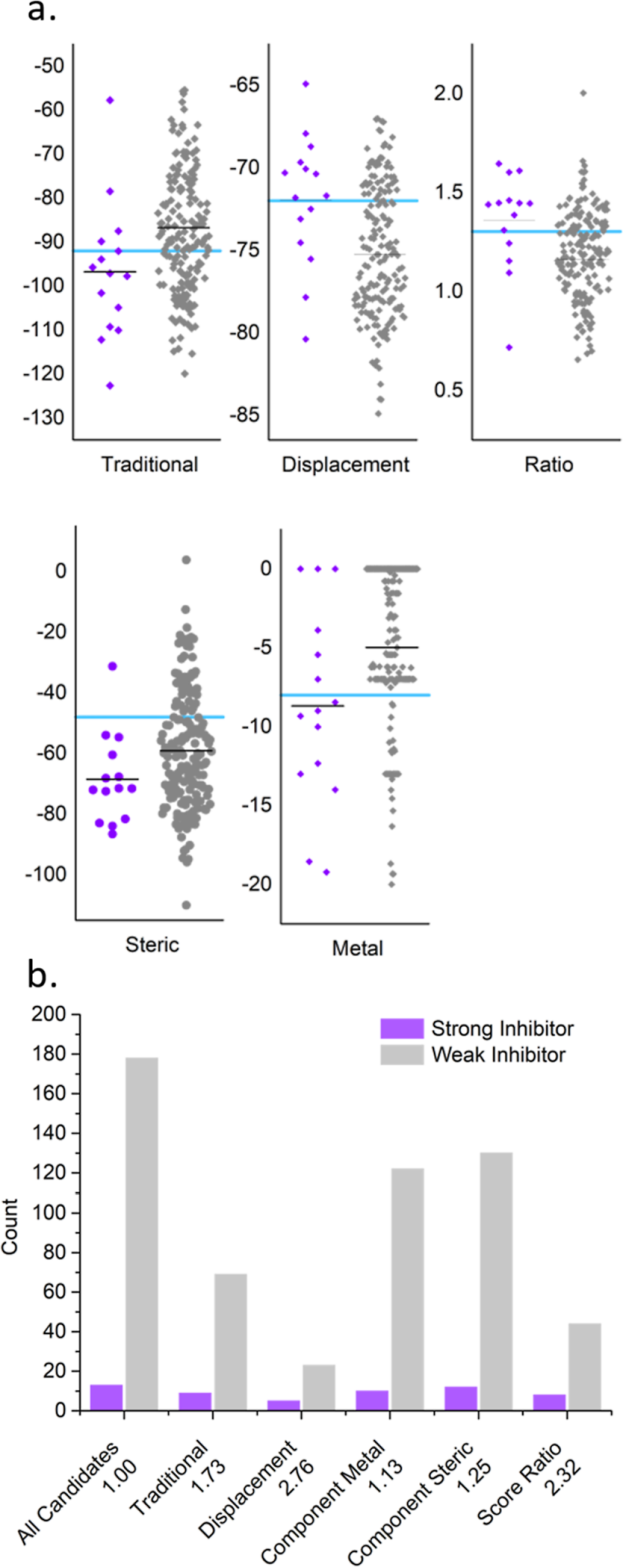
An example of substrate standardization for *D. russelii* PLA_2_. Individual substrate-based cutoff point-scoring metrics (a), including: the traditional direct docking score (obtained with *chemPLP*),^29^ our displacement docking score (this work), our combined ratio score (eq 1, this work), and steric and metal interaction scores obtained from the traditional direct docking with the *chemPLP* scoring function (see Tables S6 and S7). In these figures, the substrate-based cutoff is shown as a blue line, with the mean of each data set indicated by a black line. Comparison of the total number of compounds (all candidates) and the counts of candidate inhibitors that pass the substrate-based cutoff (b), for each metric listed (separated for strong and weak inhibitors) and with calculated enrichment (eq 2) indicated at the *x*-axis labels.

In this equation, Positive is the number of true positives and Total the total number of compounds, for either all compounds (subscript all) or the subset of compounds for which a score or scoring component surpasses the score for **S**^**M**^ (Figure 7a, blue lines). Using the direct and displacement docking results for candidate inhibitors, we could compute the enrichment from the substrate-based cutoff of every metric which we visualize alongside each metric’s results from either strong or weak inhibitors in Figure 7.

By applying the substrate-based cutoffs individually, subsets are selected with a greater proportion of strong inhibitors, which enables quantification of the performance of each metric by comparing the relative enrichment or increase in the proportion of strong inhibitors compared to the candidate population (Figure 7b). Displacement scoring, which measures active site obstruction, achieved the highest enrichment (2.76), followed by the ratio score (eq 1; 2.32), with both outperforming traditional direct docking (1.76). Since all three methods retained a similar number of strong candidates, the effectiveness of each cutoff reflects its ability to eliminate weaker candidates from the pool. Surprisingly, we found that our intuitive cutoff metrics based on metal and steric interactions—both critical to catalysis and substrate binding—yielded only modest enrichments (1.13 and 1.25).

Using this cutoff approach, we can reduce the complexity of docking scores while retaining specific assessment of inhibitor performance against individual targets that would be lost in a simple numerical aggregation (e.g., an average, cf. Figure 7a). For this purpose we consider each cutoff metric as a binary classification, awarding a point for each metric passed as shown in eq 3, where score is a dock scoring function evaluated with *ChemPLP* and ratio is determined from eq 1, and lig and subscripts dir and disp refer to the ligand and to direct and displacement docking, respectively.
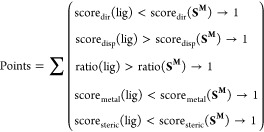
3

**Figure 8:**
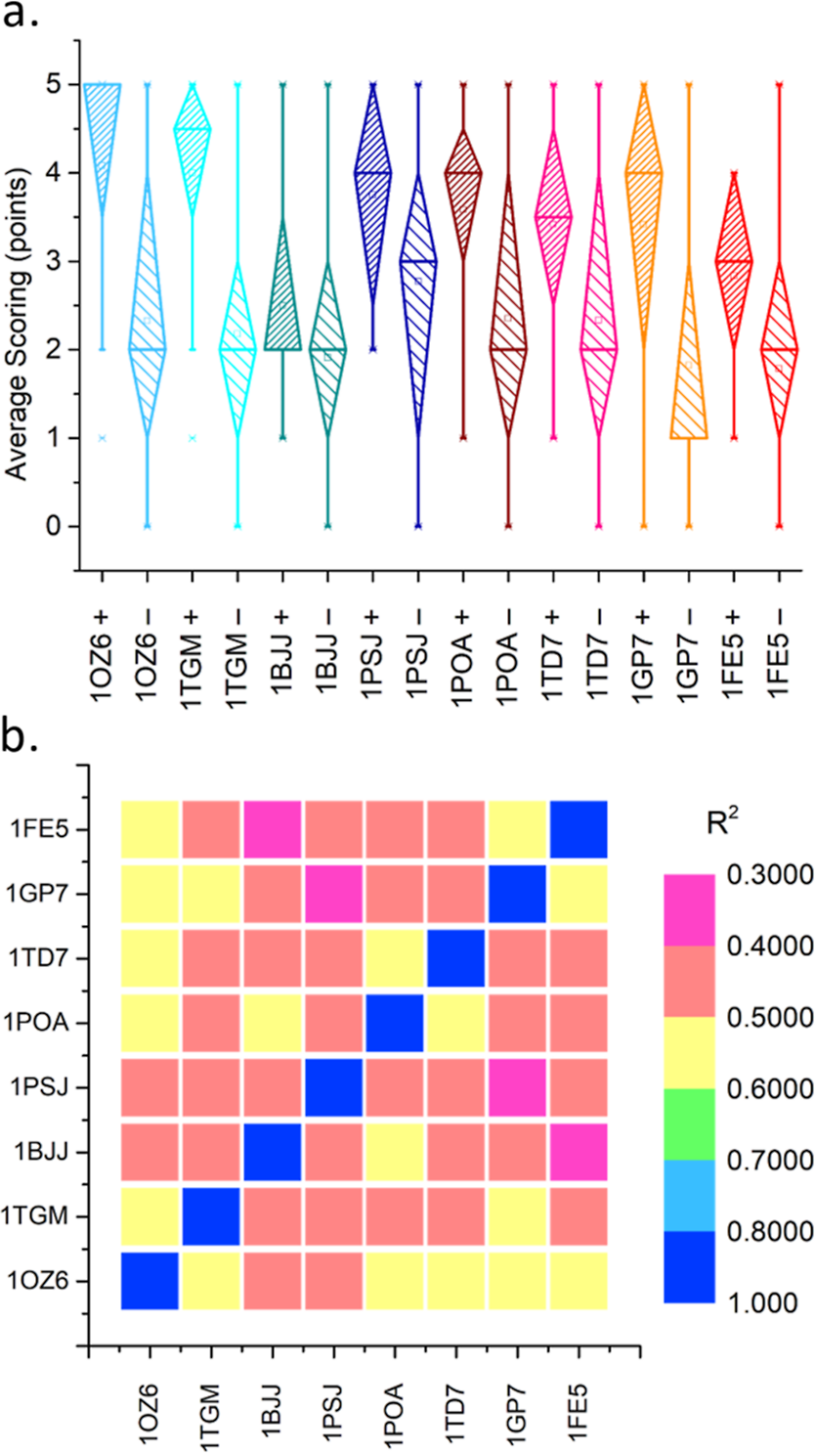
Multispecies screening results: (a) diamond boxplots of points-based scoring (eq 3) of strong (“+”) and weak inhibitors (“–”) against viper (blues) and elapid (reds) PLA_2_s (Tables S7–S14), and (b) heatmaps showing the pairwise correlation of points-based scores between inhibitors, as obtained among the PLA_2_s considered (referred to by their PDB IDs; Table 1).

From the enrichment presented by each cutoff (Figure 7b) and the intuitive theoretical basis of these cutoff metrics, we anticipate that strong inhibitors will be higher scored following our points system of eq 3. This is confirmed by our finding that strong inhibitors in our library score higher numbers of points than weak-inhibiting candidates (Figure 8a). Notably, this system is devised for identifying broad-spectrum inhibitors and is unsuitable for ordering ligands by affinity to a target due to the lack of granularity in the binary metric.

The basis for developing a small molecule inhibitor for diverse PLA_2_s leverages their structural similarity (Figure 2). With the evident predictive power of our computational method, we assessed if strong inhibitors of *D. russelii* PLA_2_ would perform similarly against isoforms from the other viper and elapid species. For this investigation we used our displacement docking, substrate standardization, and points-based scoring methods to classify the strong and weak candidates of our library, across the eight PLA_2_ used for structural comparison. Doing this indicated broad-spectrum efficacy of the inhibitors (Figure 8a), as well as similarities between PLA_2_s through predicted binding affinities (Figure 8b).

Overall, the strong inhibitors experimentally identified against *D. russelii* PLA_2_ activity scored 1–2 points higher for binding to most other PLA_2_s compared to the pool of weak inhibitors (Figure 8a). This broad predicted binding suggests that successful inhibitors may strongly interact with conserved structural features of the active site, supporting a broad inhibitory mechanism. Notable exceptions are *G. halysA* PLA_2_ (1BJJ) and *Bungarus caeruleus* (1FE5), both of which function as presynaptic neurotoxins in addition to their catalytic activity. When comparing the scores of viper and elapid species, we observe a slight reduction in the aggregate of elapid scores (1POA, 1TD7, 1GP7, 1FE5) in line with the dissimilarity between the viper (including *D. russelii*) and elapid families of snakes (Figure 2b). Taken together these results highlight both the potential and limitations of computational approaches in the discovery of broad-spectrum inhibitors against venom PLA_2_s.

Since structural similarities may reflect similar inhibitor binding, we correlated the inhibitor scores between each PLA_2_ pair to identify more similar structures and inform future experimental design (Figure 8b). Although most correlations are weak (*R*^2^ < 0.50), inhibitor affinity to 1OZ6 (*Echis carinatus*) is remarkably better correlated to both viper and elapid isoforms (*R*^2^ ≈ 0.52). Importantly, this analysis is independent of inhibitor strength or classification and could be used to broadly compare enzyme structures using diverse ligands with a wide range of expected activity. While this similarity may not directly support the discovery of broad-spectrum inhibitors, it provides unique insight into the similarity of different enzymes in inhibitor binding. It may prove beneficial for informing experimental design against isoforms that are better representative of a broader range of targets and open new avenues for computational chemistry and virtual screening in drug discovery.

## Conclusion and Outlook

In this study, we developed a set of computational tools aimed at improving the discovery of broad-spectrum inhibitors for venom PLA_2_s, as part of broader efforts to combat the neglected tropical disease of snakebite envenoming. This toolbox includes new approaches that account for enzyme structural variation, introducing displacement docking as a method to assess active site obstruction and a substrate-based standardized scoring system to evaluate inhibitor performance across different enzyme isoforms.

Using a library of molecules with experimentally known inhibitory activity against *D. russelii* PLA_2_, we found that displacement docking provided modest improvement over traditional affinity-based approaches in identifying strong inhibitors. Furthermore, combining both methods significantly enhanced classification accuracy, offering a comprehensive approach to discover strong inhibitors. While this study was aimed at a single enzyme with a shallow substrate-binding cleft, in future studies we will assess the full potential of displacement docking toward inhibitor discovery against inhibitors with diverse active site structures, and explore the development of enzyme-specific scoring functions based on substrate activation trajectories. Our substrate standardization approach facilitated cross-species comparisons, highlighting additional therapeutic targets for snakebite treatment and suggesting broader relevance of experimental results from a single venom source. Moreover, observed correlations in inhibitor performance across PLA_2_ isoforms suggest a promising strategy for identifying representative enzymes for streamlining future experimental design for inhibitor discovery.

Together, these advancements accelerate the development of potent PLA_2_ inhibitors and open new pathways for the design and discovery of drugs with applicability across diverse biological targets beyond venom enzymes. Moving forward, we aim to expand these methods to additional PLA_2_s by validating ab initio structure prediction tools. Ultimately these findings will be used to further our research efforts to reduce harm from other venom enzyme targets in the development of broadly acting small-molecule therapeutics to improve global health and equity.

## Data Availability

All data generated, including SMILES representation of molecular structures, are provided in the electronic Supporting Information. All software used is available without charge for noncommercial or academic uses.

## Supporting Information Available

Input files used for molecular dynamics analysis and clustering, comparison of scoring functions, comparison of SM docking poses into model PLA_2_ isoform 1TGM, and descriptions of data table headers and methods used for experimental inhibition assessment, protomer library development, pairwise PLA_2_ structural comparisons, docking method comparisons, displacement docking, and the combined standardized scoring of inhibitors against diverse PLA_2_ isoforms (PDF)
Complete data tables for experimental inhibition assessment, protomer library development, pairwise PLA_2_ structural comparisons, docking method comparisons, displacement docking, and the combined standardized scoring of inhibitors against diverse PLA_2_ isoforms (XLSX)
