# Supplementary material for: Computational Strategies for Broad Spectrum Venom Phospholipase A2 Inhibitors
Source: J Chem Inf Model. 2025 Apr 22;65(9):4593–601. doi: 10.1021/acs.jcim.5c00045 (PMC12076495; doi:10.1021/acs.jcim.5c00045)
Supplement: Supplementary file 1 — ci5c00045_si_001.pdf [file ci5c00045_si_001.pdf]

Supporting information:

Computational Strategies for Broad Spectrum Venom Phospholipase A_2 Inhibitors

David A. Poole III,[*1] Laura-Oana Albulescu,[2] Jeroen Kool,[1] Nicholas R. Casewell,[2] Daan P. Geerke[1]

[1] Department of Chemistry and Pharmaceutical Sciences, Amsterdam Institute for Molecular and Life Sciences, Vrije Universiteit Amsterdam, De Boelelaan 1105, Amsterdam 1081 HV, the Netherlands. *E-mail: d.a.poole@vu.nl

[2] Centre for Snakebite Research & Interventions, Liverpool School of Tropical Medicine, Pembroke Place, Liverpool, L3 5QA, United Kingdom

Contents:

Contents: 2
Section S1. PLA_2 Molecular dynamics and structure clustering 3
Table S1 3
Section S2. Comparison of S^M-PLA_2 complexes 4
Figure S1 4
Section S3. Pairwise protein sequence and structure comparison 5
Table S2 5
Section S4. Experimental dataset on *Daboia russelii* PLA_2 inhibition 6
Table S3 6
Section S5. Expanded protomer library development 7
Table S4 7
Section S6. Scoring function comparison 8
Table S5 8
Section S7. Direct and displacement docking of *Daboia russelii* PLA_2 inhibitors 9
Table S6 9
Section S8. Combined scoring results for individual PLA_2s 10
Table S7 10
Section S9. Analysis of Inhibitor Drug Likeness: 11
Figure S2 11
Section S10. References 12

Section S1. PLA_2 Molecular dynamics and structure clustering

Crystallographic structures for each PLA_2 (1OZ6, 1TGM, 1BJJ, 1PSJ, 1POA, 1TD7, 1GP7, 1FE5)[1–8] were downloaded and processed with pdb4amber to remove water, cosolvent, and ligand molecules.[9] Using tleap,[9] the processed structures were solvated with a cubic box of tip3p model water extending 10 Angstrom from the protein edge,[10] neutralized by the automated addition of Na^+ and Cl^- ions, and adjusted by the addition of 10 Na^+ and Cl^- for salinity. These models were then submitted to a standard molecular dynamics scheme with steps for minimization (100 steps CPU, with 5000 steps GPU), heating (5 ns), NPT equilibration (2 steps, 10 ns each), and NVT molecular dynamics (100 ns).[11] For this process the random seed was set to the execution time (*ig* =-1) and a 12 Angstrom cutoff used for non-bonded interactions. All molecular dynamics simulations were coupled to a Langevin thermostat (*ntt* = 3) with a minimal collision frequency (*gamma_ln* < 1.0) to minimize system artefacts in temperature management. For equilibration steps, the system was coupled to a Monte Carlo barostat at 1 atmosphere pressure. Furthermore, the parameters for each step are provided below:

Table S1: Parameters used for Molecular Dynamics

Minimzation CPU (pme_mn_cpu.in)	Minimzation GPU (pme_mn_gpu.in)	Heating (pme_ht.in)	Equilibration (2x) (pme_eq.in)	Production (pme_pro.in)
&cntrl	&cntrl	&cntrl	&cntrl	&cntrl
ntb=1,	ntb=1,	ig = -1,	ig = -1,	imin=0,
imin=1,	imin=1,	imin = 0,	imin = 0,	irest=1,
irest=0,	irest=0,	irest = 0,	irest = 1,	ntx=5,
ntx=1,	ntx=1,	ntx = 1,	ntx = 7,	nstlim=5000000,
maxcyc=100,	maxcyc=5000,	ntb = 1,	ntb = 2,	dt=0.002,
ncyc=10,	ncyc=250,	ntp = 0,	ntp = 1,	ntc=2,
ntpr=1,	ntpr=1,	cut =12,	barostat=2,	ntf=2,
ntwx=0,	ntwx=0,	ntr = 0,	pres0 = 1.00,	ig=-1,
ig= -1,	ig= -1,	ntc = 2,	taup = 0.1,	cut=12.0,
cut =12,	cut =12,	ntf = 2,	cut =12,	ntb=1,
ntpr=20,	ntpr=500,	tempi = 0.0,	ntr = 0,	ntp=0,
ntxo=2	ntxo=2	temp0 = 300.0,	ntc = 2,	ntpr=1000,
		ntt = 3,	ntf = 2,	ntwx=1000,
		gamma_ln=0.001,	tempi = 300.0,	ntt=3,
		iwrap = 1,	temp0 = 300.0,	gamma_ln=0.1,
		nstlim= 500000,	ntt = 3,	temp0=300.0,
		dt = 0.001,	gamma_ln=0.1,	ioutfm=1,
		nscm = 50000,	iwrap = 1,	iwrap=1
		ntpr = 50000,	nstlim= 500000,	
		ntwx = 50000,	dt = 0.002,	
		ntwr = 50000,	nscm = 10000,	
		ntxo=2	ntpr = 10000,	
			ntwx = 10000,	
			ntwr = 1000,	
			ntxo=2	

The resulting molecular dynamics trajectories were then clustered with cpptraj,[9] using the hierarchal agglomeration of the alpha carbon backbone positions with Epsilon value of 3.0 to produce 10 clusters and their centroid representations. The representations from the 5 most abundant clusters were then used for molecular docking (Figure 2a).

Section S2. Comparison of S^M-PLA_2 complexes

During testing of docking using the *vinardo* scoring function (smina)[12] and *ChemPLP* (PLANTS1.2),[13] we qualitatively assessed the interactions for poses generated for complexes of our model substrate S^M with *Daboia russelii* PLA_2 (1TGM).[1] This assessment was based on: 1) the distance and angle between the substrate's cleavage site and the PLA_2 histidine with the assumption of water occupancy (Figure S1, red dashes), and 2) the distance between the activated carbonyl of the cleavage site and the calcium ion center (Figure S1b, yellow dashes). From this assessment, we found that interactions in the complexes generated by *ChemPLP*/PLANTS1.2 were more consistent with substrate activation.

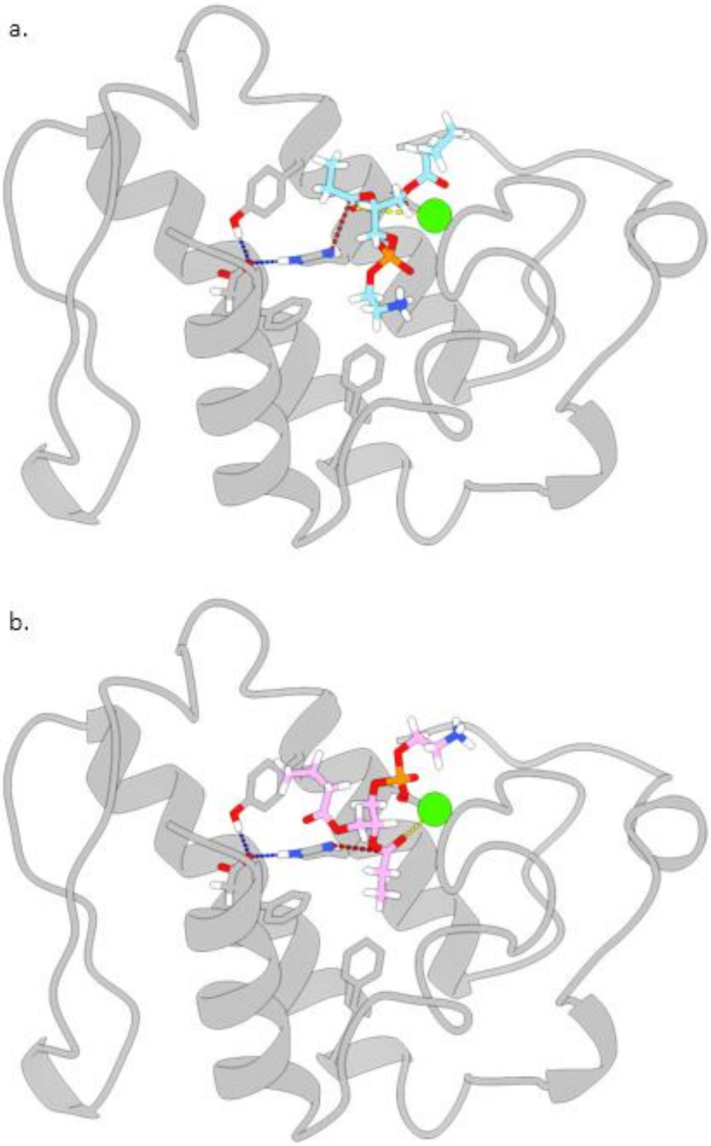

Figure S1: *Docked pose of S^M into the active site of D. russelii PLA_2 (1TGM) using (a) the vinardo scoring function implemented with smina, or (b) the ChemPLP scoring function implemented within PLANTS1.2. Interactions between the substrate and PLA_2 histidine or calcium ion center that differ between these docked poses are shown by respective red or yellow dashed lines.*

Section S3. Pairwise protein sequence and structure comparison

Pairwise comparison of PLA_2 structures from molecular dynamics were produced using a simple bash script that excluded self-comparison with a simple conditional statement. For MaxCluster,[14] default options were used with the *-id* flag to enable sequence-independent analyses between dissimilar PLA_2s. For accessibility, the full results are provided in Table S2 as an excel extended (xlsx) format file in the electronic supporting information under the tab “Table S2”. The data here include the 2450 pairwise comparisons of all PLA_2s in this study in the formulation of Figure 2b. For ease of access, the complete results are provided in Table S2 as an excel (xlsx) format file in the electronic supporting information. As an example of these data, an extract of 10 entries is shown below.

Table S2: Pairwise PLA_2 structure Comparison Results (Entries 1 – 10)

Enzyme 1	Pose 1	Enzyme 2	Pose 2	Pairs	RMSD	MAXSUB	Len	gRMSD	TM	%ID	Families
1bjj	1	1bjj	2	122	1.083	0.938	122	1.087	0.941	100	V-V
1bjj	1	1bjj	3	122	0.88	0.955	122	0.884	0.958	100	V-V
1bjj	1	1bjj	4	122	0.911	0.952	122	0.915	0.955	100	V-V
1bjj	1	1bjj	5	122	0.828	0.96	122	0.832	0.962	100	V-V
1bjj	1	1fe5	1	109	1.47	0.824	114	1.749	0.85	40.4	V-E
1bjj	1	1fe5	2	107	1.459	0.808	114	1.827	0.842	40.4	V-E
1bjj	1	1fe5	3	107	1.402	0.816	114	1.84	0.848	40.4	V-E
1bjj	1	1fe5	4	109	1.458	0.824	114	1.776	0.849	40.4	V-E
1bjj	1	1fe5	5	107	1.379	0.818	114	1.775	0.851	40.4	V-E

Legend

Column Heading	Explanation
Enzyme 1, Enzyme 2	The names of the enzymes being compared to each other by MaxCluster in entry order
Pose 1, Pose 2	The respective pose numbers of each enzyme model in entry order
Pairs	The number of aligned pairs in the sequence matching
RMSD	The positional root-mean-squared difference of the peptide backbones of the two enzymes.
MAXSUB	The MAXSUB score, a metric similar to TM-score but less widely used. In this study, the MAXSUB scores are highly correlated to the TM-scores (data not shown).
Len	The length of the aligned matching amino acid residues.
gRMSD	The generalized-RMSD, uses imputed values for a robust comparison of dissimilar structures
TM	The template modelling score as implemented by Zhang. Here the average score is used so the results are invariant to the structure ordering.
%ID	The overall identity match in primary peptide sequence calculated by MaxCluster.
Families	Identification of the two enzyme’s membership in viper (V) or elapid (E) families.

Section S4. Experimental dataset on *Daboia russelii* PLA_2 inhibition

This table includes all candidate inhibitors used in initial high-throughput screening for inhibition of *Daboia russelii* venom PLA_2 activity. This enzymatic assay compares the rate of a model substrate disappearance in treated (the addition of a fixed concentration of inhibitor) versus untreated (with the addition of a blank) control samples.[15] Due to the variances in measurement conditions, some entries may have an inhibition less than 0% (i.e., the enzyme activity was increased relative to control), or greater than 100% (i.e., final substrate concentration was higher than control).

Each ligand was initially provided with a SMILES formatted string for the chemical structure of the absolute formula as listed on public databases and these SMILES strings were manually reprocessed to remove minor fragments and salt components derived from the specific formulations so that a hydrochloride salt of a drug would be processed into its free base form. The resulting SMILES strings were then processed with openbabel[16] for standardization as a canonical SMILES and to generate a corresponding INCHI as a simplified chemical identifier.

For ease of access, the complete results are provided in Table S3 as an excel (xlsx) format file in the electronic supporting information. As an example of these data, an extract of 10 entries is shown below.

Table S3: Experimental Dataset of *Daboia Russelii* PLA_2 Inhibitors (Entries 1–10)

Ligand INCHI Key	Ligand Name	Percent Ligand Inhibition
BHLXTPHDSZUFHR-UHFFFAOYSA-N	Varespladib	100.46
PXRCDPVYRPKGGI-MRXNPFEDSA-N	AZD2716	100.00
OPWQYOUZRHDKBR-UHFFFAOYSA-N	LY 311727	99.27
DTGKSKDOIYIVQL-SHTILUHOSA-N	D-Borneol	99.24
VJYDOJXJUCJUHL-UHFFFAOYSA-N	Varespladib methyl	98.72
LRBQNJMCXXYXIU-NRMVVENXSA-N	Tannic Acid	98.30
REUKAAAAEHWGGM-UHFFFAOYSA-N	ROC-0929	91.70
DTGLZDAWLRGWQN-UHFFFAOYSA-N	Prasugrel	89.92
QBKSWRVVCFFDOT-UHFFFAOYSA-N	Gossypol	87.39
YMGFTDKNIWPMGF-UCPJVGPRSA-N	Salvianolic acid	87.11

Legend

Column Heading	Explanation
Ligand INCHI Key	The INCHI key used to represent the ligand and its derivative forms throughout analyses
Ligand Name	The common name assigned to the ligand, most of which are internal or trade names.
Percent Ligand Inhibition	The experimental inhibitor strength from high throughput screening, in percent activity compared to untreated *Daboia russelii* venom samples

Section S5. Expanded protomer library development

Using the initial SMILES representation of each ligand structure a series of protomers were generated using Dimorphite-DL[17] for a pH range between 8.0 and 5.0 since the catalytic action of PLA_2 yields increasing fatty acid concentration and concomitant decrease in the environment's pH. While this approach could be exhaustive, the outputs were limited to a maximum of 8 protomers per ligand using the--max-variants flag, which accommodated most ligands in initial testing with notable exceptions including tannic acid which feature 2^{25} protomeric states that are readily accessible at this pH range. Overall, this limitation is necessary to reduce the computational workload and to minimize biasing induced by the increased sampling of ligands with more protomers. For ease of access, the complete results are provided in Table S4 as an excel (xlsx) format file in the electronic supporting information. As an example of these data, an extract of 10 entries is shown below.

Table S4: Expanded Protomer Library Dataset (Entries 1–10)

Ligand Base INCHI Key	Ligand Protomer Number	MW	LogP (obabel)	Positive Charges	Negative Charges	Number Charges	Net Charge	HBA	HBD	Lipinski's Criteria	Ligand Protomer SMILES
AEXFXNFMSAAELR-RXVVDRJESA-N	0	420.461	3.02	1	0	1	1	8	2	4	C[N+]1C(=O)Oc2ccc(c3ccc(C[C@@H](C#N)NC(=O)[C@@H]4CNCCCO4)cc3)cc12
AEXFXNFMSAAELR-RXVVDRJESA-N	1	421.469	3.23	2	0	2	2	7	2	4	C[N+]1C(=O)Oc2ccc(c3ccc(C[C@@H](C#N)NC(=O)[C@@H]4C[NH2+]CCCO4)cc3)cc12
AFCGFAGUEYAMAO-UHFFFAOYSA-N	0	180.202	0.10	0	3	3	-3	5	1	4	CC(=O)NCCCS([O-])([O-])[O-]
AGPIHNZOZNKRGT-CYBMUJFWSA-N	0	455.906	5.10	1	1	2	0	4	1	3	O=C(c1cc(S(=O)(=O)[N-]c2ccccc2F)c(F)cc1Cl)N1CC[N@H+]2CCC[C@@H]2C1
AGPIHNZOZNKRGT-CYBMUJFWSA-N	1	457.922	4.80	2	0	2	2	4	3	4	O=C(c1cc(S(=O)(=O)Nc2ccccc2F)c(F)cc1Cl)[NH+]1CC[N@@H+]2CCC[C@@H]2C1
AGPIHNZOZNKRGT-CYBMUJFWSA-N	2	455.906	5.10	1	1	2	0	4	1	3	O=C(c1cc(S(=O)(=O)[N-]c2ccccc2F)c(F)cc1Cl)[NH+]1CC[N@@]2CCC[C@@H]2C1
AGPIHNZOZNKRGT-CYBMUJFWSA-N	3	455.906	4.37	0	0	0	0	6	1	4	O=C(c1cc(S(=O)(=O)Nc2ccccc2F)c(F)cc1Cl)N1CC[N@@]2CCC[C@@H]2C1
AGPIHNZOZNKRGT-CYBMUJFWSA-N	4	456.914	4.58	1	0	1	1	5	2	4	O=C(c1cc(S(=O)(=O)Nc2ccccc2F)c(F)cc1Cl)[NH+]1CC[N@@]2CCC[C@@H]2C1
AGPIHNZOZNKRGT-CYBMUJFWSA-N	5	456.914	4.58	1	0	1	1	5	2	4	O=C(c1cc(S(=O)(=O)Nc2ccccc2F)c(F)cc1Cl)N1CC[N@@H+]2CCC[C@@H]2C1
AGPIHNZOZNKRGT-CYBMUJFWSA-N	6	456.914	5.32	2	1	3	1	3	2	3	O=C(c1cc(S(=O)(=O)[N-]c2ccccc2F)c(F)cc1Cl)[NH+]1CC[N@@H+]2CCC[C@@H]2C1
AGPIHNZOZNKRGT-CYBMUJFWSA-N	7	454.898	4.89	0	1	1	-1	5	0	4	O=C(c1cc(S(=O)(=O)[N-]c2ccccc2F)c(F)cc1Cl)N1CC[N@]2CCC[C@@H]2C1

Legend

Column Heading	Explanation
Ligand Base INCHI Key	The key used to represent the ligand from which the protomer is derived, see Table S2c.
Ligand Protomer Number	An index number to refer to each protomer with 0 being found at the highest pH.
MW	Protomer molecular weight as a proxy for inhibitor molecular size.
LogP (obabel)	The octanol-water partitioning coefficient computed using open babel.
Positive Charges	The number of positive charges in the protomer.
Negative Charges	The number of negative charges in the protomer.
Number Charges	The absolute total number of charges in the protomer.
Net Charge	The sum of charges in the protomer.
HBA	The number of hydrogen bond acceptors identified by open babel.
HBD	The number of hydrogen bond donors identified by open babel.
Lipinski's Criteria	The total number of Lipinski criteria satisfied by the protomer.
Ligand Protomer SMILES	The SMILES representation of each ligand protomer.

Section S6. Scoring function comparison

The parameters for both smina and PLANTS1.2 were chosen to produce the most reliable and best possible scoring while limiting the analysis of each protomer to a reasonable computational cost for large scale analysis. For the purpose of this screening, we limited the extent of docking parameters to limit docking runs to 30 CPU-seconds on average. In preparation for docking all PLA_2 models were aligned and positioned using ChimeraX so the calcium ion was at the origin point.[18]

For smina, the default options for exhaustiveness were chosen since initial testing showed little improvement on higher values. The initial docking site was selected as a 15-Angstrom cube centered on the calcium ion (position = 0, 0, 0). To accommodate larger ligands, the docking site was allowed to expand as needed without penalty.

For PLANTS1.2, the binding site was defined as a 10-Angstrom sphere about the origin point (position = 0, 0, 0), and several optional parameters were enabled relating to metal binding and steric contributions (enable_sulphur_acceptors = 1, ligand_clash_include_HH = 1, ligand_intra_score lj). In addition, ligand flexibility was enabled as well as similar considerations regarding ligand flexibility (flip_amide_bonds = 1, flip_ring_corners = 1, force_planar_bond_rotation = 1, force_flipped_bonds_planarity = 1, flip_planar_n = 1). In order to accommodate larger ligands outside_binding_site_penalty parameter was set to 0.0.

For both methods, all ligands and their protomers were docked against all five poses for the PLA_2 1TGM, with the best score across all ligand/protomers used for further analysis including the composition of Figure 4a. For ease of access, the complete results are provided in Table S5 as an excel (xlsx) format file in the electronic supporting information. As an example of these data, an extract of 10 entries is shown below.

Table S5: Dataset of Inhibitor-1TGM Affinity Scores Derived from Diverse Docking Functions (Entries 1–10)

Scoring Function	Enzyme Name	Enzyme Pose	Ligand Base INCHI Key	Ligand Protomer	Score
ad4	1tgm	1	AEXFXNFMSAAELR-RXVVDRJESA-N	0	-36.32
ChemPLP	1tgm	1	AEXFXNFMSAAELR-RXVVDRJESA-N	0	-81.80
dkoes	1tgm	1	AEXFXNFMSAAELR-RXVVDRJESA-N	0	-5.49
PLP	1tgm	1	AEXFXNFMSAAELR-RXVVDRJESA-N	0	-72.13
vina	1tgm	1	AEXFXNFMSAAELR-RXVVDRJESA-N	0	-8.55
vinardo	1tgm	1	AEXFXNFMSAAELR-RXVVDRJESA-N	0	-7.10
ChemPLP	1tgm	2	AEXFXNFMSAAELR-RXVVDRJESA-N	0	-88.00
dkoes	1tgm	2	AEXFXNFMSAAELR-RXVVDRJESA-N	0	-5.54
PLP	1tgm	2	AEXFXNFMSAAELR-RXVVDRJESA-N	0	-73.15
vina	1tgm	2	AEXFXNFMSAAELR-RXVVDRJESA-N	0	-7.64

Legend

Column Heading	Explanation
Scoring Function	The scoring function used in this analysis.
Enzyme Name	The enzyme name used in this analysis.
Enzyme Pose	The number of the enzyme pose used in this analysis.
Ligand Base INCHI Key	The key used to represent the ligand from which the protomer is derived, see Table S2c.
Ligand Protomer	An index number to refer to each protomer with 0 being found at the highest pH.
Score	The best affinity score obtained from the poses generated during the docking simulation

Section S7. Direct and displacement docking of *Daboia russelii* PLA_2 inhibitors

Traditional and displacement docking (using PLANTS and settings described in Section 5 of this supporting information) were automated with a bash script that managed the input and output file naming by the modification of the protein_file and ligand_file fields of a default input file. For displacement docking the standard input file would be modified by the addition of the shape_constraint field that specified the best pose generated by traditional docking with an assigned score of 100 (i.e., a very strong penalty). This penalty well exceeds the maximum traditional docking scores observed for the substrates in any PLA_2 (see Tables S7 – S14), thereby excluding co-occupation of the substrate in to the ligand-occupied space. Moreover, while this approach was carried out for each ligand protomer against each enzyme pose, the scores here are reported for only the protomer-pose combination with the best (i.e. most negative) score for traditional (direct) docking (i.e., the best docked ligand). This data coordination could lead to some biasing of the displacement scores, however there is no clear correlation between the results found in traditional and displacement docking approaches as shown in Figure 6c.

For ease of access, the complete results are provided in Table S6 as an excel (xlsx) format file in the electronic supporting information. As an example of these data, an extract of 10 entries is shown below.

Table S6: Traditional (Direct) and Displacement Docking Scores (Entries 1–10)

Ligand Base INCHI Key	Ligand Name	Traditional Docking	Displacement Score	Ratio
AEXFXNFMSAAELR-RXVVDRJESA-N	Brensocatib	-93.27	-77.97	1.20
AFCGFAGUEYAMAO-UHFFFAOYSA-N	Acamprosate	-59.82	-78.59	0.76
AGPIHNZOZNKRGT-CYBMUJFWSA-N	ABT-639	-85.87	-69.59	1.23
AIWRTTMUVOZGPW-HSPKUQOVSA-N	Abarelix	-11.52	-79.74	0.14
ALWKGYPQUAPLQC-UHFFFAOYSA-N	Neostigmine	-63.39	-79.57	0.80
ANZXOIAKUNOVQU-UHFFFAOYSA-N	Bambuterol	-92.64	-68.74	1.35
ASOADIZOVZTJSR-UHFFFAOYSA-N	Opicapone	-95.89	-75.39	1.27
AVOLMBLBETYQHX-UHFFFAOYSA-N	Ethacrynic acid	-75.03	-75.73	0.99
BAZRWWGASYWYGB-SNVBAGLBSA-N	GDC-0575	-72.39	-73.82	0.98
BCFGMOOMADDAQU-UHFFFAOYSA-N	Lapatinib	-109.00	-75.51	1.44

Legend

Column Heading	Explanation
Ligand Base INCHI Key	The key used to represent the ligand from which the protomer is derived, see Table S2c.
Ligand Name	The name of the ligand, included for convenience.
Traditional Docking	The *ChemPLP* score of the best docking pose (i.e., the most negative score) found for all protomers of the ligand against all poses of the 1TGM by PLANTS1.2 (unit: …)
Displacement Score	The *ChemPLP* score of the best docking pose (i.e., the least negative score) for S^M when docked against the 1TGM pose with the best Traditional Docking score and shape constraint corresponding to that specific docked protomer structure such that consistent pose-protomers pairs are used.
Ratio	The Traditional Docking score denominated by the Displacement Score (see Equation 2).

Section S8. Combined scoring results for individual PLA_2s

Traditional and displacement docking (using PLANTS and settings described in Section 5 of this supporting information) were automated with a bash script that managed the input and output file naming by the modification of the protein_file and ligand_file fields of a default input file. For displacement docking the standard input file would be modified by the addition of the shape_constraint field that specified the best pose generated by traditional docking with an assigned score of 100 (i.e., a very strong penalty). This penalty well exceeds the maximum traditional docking scores observed for the substrates in any PLA_2 (see Tables S7–S14), thereby excluding co-occupation of the substrate in to the ligand-occupied space. Moreover, while this approach was carried out for each ligand protomer against each enzyme pose, the scores here are reported for only the protomer-pose combination with the best (i.e. most negative) score for traditional (direct) docking (i.e., for the best docked ligand). Following the substrate-standardization scheme (Figure 7a), five key values are used to classify likely strong inhibitors which are converted to a binary point metric (Equation 3) based on the results for the model substrate S^M. Complete results are provided as Tables S7–S14 (separated per isoform, as indicated by the corresponding PDB ID in the separate excel-tab names for the tables) in an extended excel (xlsx) formatted files in the electronic supporting information. As an example of these data, an extract of 10 entries from Table S7 is shown below.

Table S7: Combined Inhibitor Scoring Results For 1TGM (Entries 1–10)

Enzyme Name	Best Enzyme Pose	Ligand Base INCHI Key	Ligand Name	Ligand Best Protomer	Score Displacement Docking	Score Direct Docking	ChemPLP Metal	ChemPLP Steric	Ratio	Displacement Point	Direct Docking Point	Metal Point	Steric Point	Ratio Point	Sum Points
1tgm	2		Model Substrate (SM)	0	-70.33	-92.05	-0.40	-48.72	1.34						
1tgm	4	AEXFXNFMSAAELR-RXVVDRJESA-N	Brensocatib	0	-77.97	-93.27	0.00	-72.56	1.20	0	1	0	1	0	2
1tgm	4	AFCGFAGUEYAMAO-UHFFFAOYSA-N	Acamprosate	0	-78.59	-59.82	-13.00	-21.72	0.76	0	0	1	0	0	1
1tgm	4	AGPIHNZOZNKRGT-CYBMUJFWSA-N	ABT-639	0	-69.59	-85.87	-6.99	-67.51	1.23	1	0	1	1	0	3
1tgm	5	AIWRTTMUVOZGPW-HSPKUQOVSA-N	Abarelix	1	-79.74	-11.52	-0.78	-57.39	0.14	0	0	1	1	0	2
1tgm	3	ALWKGYPQUAPLQC-UHFFFAOYSA-N	Neostigmine	0	-79.57	-63.39	0.00	-54.68	0.80	0	0	0	1	0	1
1tgm	2	ANZXOIAKUNOVQU-UHFFFAOYSA-N	Bambuterol	2	-68.74	-92.64	-3.89	-63.89	1.35	1	1	1	1	1	5
1tgm	4	ASOADIZOVZTJSR-UHFFFAOYSA-N	Opicapone	5	-75.39	-95.89	-6.93	-59.04	1.27	0	1	1	1	0	3
1tgm	3	AVOLMBLBETYQHX-UHFFFAOYSA-N	Ethacrynic acid	1	-75.73	-75.03	-14.01	-50.48	0.99	0	0	1	1	0	2
1tgm	4	BAZRWWGASYWYGB-SNVBAGLBSA-N	GDC-0575	0	-73.82	-72.39	-7.00	-55.88	0.98	0	0	1	1	0	2
1tgm	4	BCFGMOOMADDAQU-UHFFFAOYSA-N	Lapatinib	3	-75.51	-109.00	-1.91	-87.66	1.44	0	1	1	1	1	4

Legend

Column Heading	Explanation
Enzyme Name	The 4-letter code used for the PLA_2
Best Enzyme Pose	The number of the enzyme pose found with the best direct docking score
Ligand Base INCHI Key	The identifier of the ligand from which the protomer is derived, see Table S2c.
Ligand Name	The name of the ligand, included for convenience
Ligand Best Protomer	The number of the protomer found with the best direct docking score
Score Displacement Docking	The *ChemPLP* score of the best docking pose (i.e., the most negative score) for S^M when docked when docked against the enzyme-ligand complex with the best direct docking score.
Score Direct Docking	The *ChemPLP* score of the best docking pose (i.e., the most negative score) found for all protomers of the ligand against all poses of the 1TGM by PLANTS1.2
ChemPLP Metal	The sum of *ChemPLP* and *PLP* score metal components in the Direct Docking Score
ChemPLP Steric	The sum of *ChemPLP* and *PLP* score steric components in the Direct Docking Score
Ratio	The direct docking score denominated by the displacement score (see Equation 2).
Displacement Point	1 if the displacement docking score is greater than that for S^M
Direct Docking Point	1 if the direct docking score is less than that for S^M
Metal Point	1 if the *ChemPLP* Metal is less than that for S^M
Steric Point	1 if the *ChemPLP* Steric is less than that for S^M
Ratio Point	1 if the ratio is greater than that for S^M
Total Points	The sum of the 5 point functions listed above (see Equation 3)

Section S9. Analysis of Inhibitor Drug Likeness:

To characterize the drug-likeness of our protomer library, we used open babel it compute the estimated partitioning coefficient (*logP*), molecular weight (MW), and the number of hydrogen bond donors or acceptors for each protomer (Table S4). These values were then used to compute their passing four drug-likeness criteria following Lipinski's Rule of Five (Figure S2).

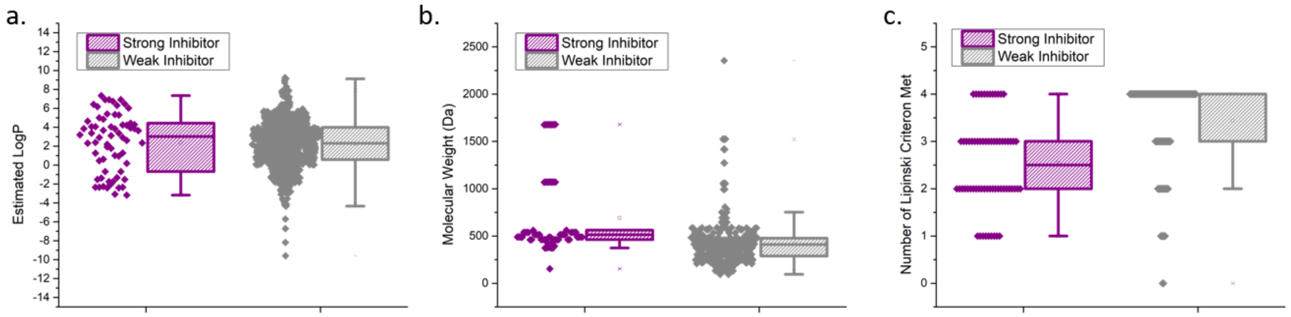

Figure S2: *Computed drug-likeness parameters including (a) estimated LogP, (b) molecular weight, and (c) Lipinski's Rule of Five criteria for strong and weak inhibitors against venom PLA_2. Strong inhibitors are defined as reducing venom PLA_2 activity by 75% at a 10µM inhibitor concentration.*

Since the initial screening library was produced with the intention of drug-repurposing to discover new venom PLA_2 inhibitors, the drug-likeness of the expanded protomer library was strongly biased towards the Lipinski's Rule of Five criteria with most inhibitors meeting at least 3 of the 4 criteria (Figure S2c). In this case, the drug-likeness of strong inhibitors was significantly lower than the overall population, suggesting that traditional drug design rationale may not be as useful for the development of venom PLA_2 inhibitors. For these protomers, the differences in estimated LogP were found to be broadly insignificant with respect to the experimental data (Figure S2a), despite the presence of more highly charged species (Figure 3). Inspection of the dataset reveals that the highly charged species correspond to relatively large poly-phenolates (MW > 500), which despite their significant charge are largely aromatic and poorly soluble under physiological conditions (pH = 7.4, T = 37°C). While the strong inhibitors were notably heavier (and thus larger), we attribute this to the bias in the dataset observed in the drug-likeness criterion (MW < 500) rather than an explainable feature of the more complex molecules.

Section S10. References

1. (a) Jasti, J.; Paramasivam, M.; Srinivasan, A.; Singh, T. P. Structure of an Acidic Phospholipase A2from Indian Saw-Scaled Viper (*Echis Carinatus*) at 2.6 Å Resolution Reveals a Novel Intermolecular Interaction. *Acta. Crystallogr. D. Biol. Crystallogr.*, **2003**, 60, 66–72. DOI: 10.1107/s090744490302208x. (b) Jasti, J.; Paramasivam, M.; Srinivasan, A.; Singh, T. P. X-Ray Structure of Acidic Phospholipase A2 from Indian Saw-Scaled Viper (*Echis Carinatus*) with a Potent Platelet Aggregation Inhibitory Activity, **2003**. DOI: 10.2210/pdb1oz6/pdb

2. Singh, N.; Jabeen, T.; Sharma, S.; Bhushan, A.; Singh, T. P. Crystal Structure of a Complex Formed between Group II Phospholipase A2 and Aspirin at 1.86 A Resolution, **2004**. DOI: 10.2210/pdb1tgm/pdb.

3. (a) Tang, L.; Zhou, Y.-C.; Lin, Z.-J. Structure of Agkistrodotoxin in an Orthorhombic Crystal Form with Six Molecules per Asymmetric Unit. *Acta. Crystallogr. D. Biol. Crystallogr.*, **1999**, 55, 1986–1996. DOI: 10.1107/s0907444999012603. (b) Tang, L.; Zhou, Y.; Lin, Z. AGKISTRODOTOXIN, A PHOSPHOLIPASE A2-TYPE PRESYNAPTIC NEUROTOXIN FROM AGKISTRODON HALYS PALLAS, **1999**. DOI: 10.2210/pdb1bjj/pdb.

4. (a) Wang, X.; Yang, J.; Gui, L.; Lin, Z.; Chen, Y.; Zhou, Y. Crystal Structure of an Acidic Phospholipase A2from the Venom of Agkistrodon Halyspallas at 2.0 Å Resolution. *J. Mol. Biol.*, **1996**, 255, 669–676. DOI: 10.1006/jmbi.1996.0054. (b) Wang, X. Q.; Lin, Z. J. ACIDIC PHOSPHOLIPASE A2 FROM AGKISTRODON HALYS PALLAS, **1996**. DOI: 10.2210/pdb1psj/pdb.

5. (a) Scott, D. L.; White, S. P.; Otwinowski, Z.; Yuan, W.; Gelb, M. H.; Sigler, P. B. Interfacial Catalysis: The Mechanism of Phospholipase A2. *Science*, **1990**, 250, 1541–1546. DOI: 10.1126/science.2274785 (b) Scott, D. L.; Otwinowski, Z.; Sigler, P. B. INTERFACIAL CATALYSIS: THE MECHANISM OF PHOSPHOLIPASE A2, **1993**. DOI: 10.2210/pdb1poa/pdb.

6. (a) Jabeen, T.; Singh, N.; Singh, R. K.; Sharma, S.; Somvanshi, R. K.; Dey, S.; Singh, T. P. Non-Steroidal Anti-Inflammatory Drugs as Potent Inhibitors of Phospholipase A2: Structure of the Complex of Phospholipase A2 with Niflumic Acid at 2.5 Å Resolution. *Acta. Crystallogr. D. Biol. Crystallogr.*, **2005**, 61, 1579–1586. DOI: 10.1107/s0907444905029604. (b) Jabeen, T.; Singh, N.; Singh, R. K.; Sharma, S.; Perbandt, M.; Betzel, C.; Singh, T. P. Interactions of a Specific Non-Steroidal Anti-Inflammatory Drug (NSAID) with Group I Phospholipase A2 (PLA2): Crystal Structure of the Complex Formed between PLA2 and Niflumic Acid at 2.5 A Resolution, **2004**. DOI: 10.2210/pdb1td7/pdb.

7. (a) Zhang, H.; Xu, S.; Wang, Q.; Song, S.; Shu, Y.; Lin, Z. Structure of a Cardiotoxic Phospholipase A2 from Ophiophagus Hannah with the "Pancreatic Loop." *J. Struct. Biol.*, **2002**, 138, 207–215. DOI: 10.1016/s1047-8477(02)00022-9. (b) Zhang, H.; Lin, Z. Acidic Phospholipase A2 from Venom of Ophiophagus Hannah, **2002**. DOI: 10.2210/pdb1gp7/pdb.

8. (a) Singh, G.; Gourinath, S.; Sharma, S.; Paramasivam, M.; Srinivasan, A.; Singh, T. P. Sequence and Crystal Structure Determination of a Basic Phospholipase A2 from Common Krait (Bungarus Caeruleus) at 2.4 Å Resolution: Identification and Characterization of Its Pharmacological Sites 1 1Edited by R. Huber. *J. Mol. Biol.*, **2001**, 307, 1049–1059. DOI: 10.1006/jmbi.2001.4550. (b) Singh, G.; Gourinath, S.; Sharma, S.; Paramasivam, M.; Srinivasan, A.; Singh, T. P. SEQUENCE AND CRYSTAL STRUCTURE OF A BASIC PHOSPHOLIPASE A2 FROM COMMON KRAIT (BUNGARUS CAERULEUS) AT 2.4 RESOLUTION: IDENTIFICATION AND CHARACTERIZATION OF ITS PHARMACOLOGICAL SITES., **2001**. DOI: 10.2210/pdb1fe5/pdb.

9. David A. Case, Hasan Metin Aktulga, Kellon Belfon, David S. Cerutti, G. Andrés Cisneros, Vinícius Wilian D. Cruzeiro, Negin Forouzesh, Timothy J. Giese, Andreas W. Götz, Holger Gohlke, Saeed Izadi, Koushik Kasavajhala, Mehmet C. Kaymak, Edward King, Tom Kurtzman, Tai-Sung Lee, Pengfei Li, Jian Liu, Tyler Luchko, Ray Luo, Madushanka Manathunga, Matias R. Machado, Hai Minh Nguyen, Kurt A. O'Hearn, Alexey V. Onufriev, Feng Pan, Sergio Pantano, Ruxi Qi, Ali Rahnamoun, Ali Risheh, Stephan Schott-Verdugo, Akhil Shajan, Jason Swails, Junmei Wang, Haixin Wei, Xiongwu Wu, Yongxian Wu, Shi Zhang, Shiji Zhao, Qiang Zhu, Thomas E. Cheatham III, Daniel R. Roe, Adrian Roitberg, Carlos Simmerling, Darrin M. York, Maria C. Nagan, and Kenneth M. Merz Jr. *J. Chem. Inf. and Model.* **2023** *63* (20), 6183-6191. DOI: 10.1021/acs.jcim.3c01153

10. Jorgensen, W. L.; Chandrasekhar, J.; Madura, J. D.; Impey, R. W.; Klein, M. L. Comparison of Simple Potential Functions for Simulating Liquid Water. The Journal of Chemical Physics, 1983, 79, 926–935. DOI:10.1063/1.445869.

11. D.A. Case, H.M. Aktulga, K. Belfon, I.Y. Ben-Shalom, J.T. Berryman, S.R. Brozell, D.S. Cerutti, T.E. Cheatham, III, G.A. Cisneros, V.W.D. Cruzeiro, T.A. Darden, N. Forouzesh, M. Ghazimirsaeed, G. Giambaşu, T. Giese, M.K. Gilson, H. Gohlke, A.W. Goetz, J. Harris, Z. Huang, S. Izadi, S.A. Izmailov, K. Kasavajhala, M.C. Kaymak, A. Kovalenko, T. Kurtzman, T.S. Lee, P. Li, Z. Li, C. Lin, J. Liu, T. Luchko, R. Luo, M. Machado, M. Manathunga, K.M. Merz, Y. Miao, O. Mikhailovskii, G. Monard, H. Nguyen, K.A. O'Hearn, A. Onufriev, F. Pan, S. Pantano, A. Rahnamoun, D.R. Roe, A. Roitberg, C. Sagui, S. Schott-Verdugo, A. Shajan, J. Shen, C.L. Simmerling, N.R. Skrynnikov, J. Smith, J. Swails, R.C. Walker, J. Wang, J. Wang, X. Wu, Y. Wu, Y. Xiong, Y. Xue, D.M. York, C. Zhao, Q. Zhu, and P.A. Kollman (2024), Amber 2024, University of California, San Francisco.

12. (a) Koes, D. R.; Baumgartner, M. P.; Camacho, C. J. Lessons Learned in Empirical Scoring with Smina from the CSAR 2011 Benchmarking Exercise. *J. Chem. Inf. Model.*, **2013**, 53, 1893–1904. DOI: 10.1021/ci300604z. (b) Quiroga, R.; Villarreal, M. A. Vinardo: A Scoring Function Based on Autodock Vina Improves Scoring, Docking, and Virtual Screening. PLOS ONE, **2016**, 11, e0155183. DOI: 10.1371/journal.pone.0155183.

13. Korb, O.; Stützle, T.; Exner, T. E. Empirical Scoring Functions for Advanced Protein−Ligand Docking with PLANTS. *J. Chem. Inf. Model.*, **2009**, 49, 84–96. DOI: 10.1021/ci800298z.

14. Ortiz, A. R.; Strauss, C. E. M.; Olmea, O. MAMMOTH (Matching Molecular Models Obtained from Theory): An Automated Method for Model Comparison. *Protein Sci.*, **2002**, 11, 2606–2621. DOI: 10.1110/ps.0215902.

15. Hall, S. R.; Rasmussen, S. A.; Crittenden, E.; Dawson, C. A.; Bartlett, K. E.; Westhorpe, A. P.; Albulescu, L.-O.; Kool, J.; Gutiérrez, J. M.; Casewell, N. R. Repurposed Drugs and Their Combinations Prevent Morbidity-Inducing Dermonecrosis Caused by Diverse Cytotoxic Snake Venoms. *Nat Comm*, **2023**, 14. DOI: 10.1038/s41467-023-43510-w

16. O'Boyle, N. M.; Banck, M.; James, C. A.; Morley, C.; Vandermeersch, T.; Hutchison, G. R. Open Babel: An Open Chemical Toolbox. Journal of Cheminformatics, 2011, 3. https://doi.org/10.1186/1758-2946-3-33.

17. Ropp, P. J.; Kaminsky, J. C.; Yablonski, S.; Durrant, J. D. Dimorphite-DL: An Open-Source Program for Enumerating the Ionization States of Drug-like Small Molecules. *J. Chem. Inf. Model.*, **2019**, 11. DOI: 10.1186/s13321-019-0336-9.

18. Meng, E. C.; Goddard, T. D.; Pettersen, E. F.; Couch, G. S.; Pearson, Z. J.; Morris, J. H.; Ferrin, T. E. UCSF ChimeraX: Tools for Structure Building and Analysis. Protein Science, 2023, 32. https://doi.org/10.1002/pro.4792.
